# Explorations on the ecological role of toxin secretion and delivery in jawless predatory Polychaeta

**DOI:** 10.1038/s41598-018-26031-1

**Published:** 2018-05-16

**Authors:** N. Cuevas, M. Martins, A. P. Rodrigo, C. Martins, P. M. Costa

**Affiliations:** 1 UCIBIO – Research Unit on Applied Molecular Biosciences, Departamento de Ciências da Vida, Faculdade de Ciências e Tecnologia da Universidade Nova de Lisboa, 2829-516 Caparica, Portugal; 2 MARE – Marine and Environmental Sciences Centre, Departamento de Ciências e Engenharia do ambiente, Faculdade de Ciências e Tecnologia da Universidade Nova de Lisboa, 2829-516 Caparica, Portugal; 3 UCIBIO – Research Unit on Applied Molecular Biosciences, Departamento de Química Faculdade de Ciências e Tecnologia da Universidade Nova de Lisboa, 2829-516 Caparica, Portugal

## Abstract

Motivated by biotechnological prospects, there is increasing evidence that we may just be scraping the tip of the iceberg of poisonous marine invertebrates, among which the Polychaeta are promising candidates for bioprospecting. Here we show that an inconspicuous phyllodocid uses toxins in its uncanny feeding strategy. The worm, a jawless active predator characterised by its bright green colour, preys on larger invertebrates (including conspecifics) by extracting tissue portions with its powerful proboscis through suction. The animal is even able to penetrate through the valves and plates of live molluscs and barnacles. Observations *in situ* and a series of experiments demonstrated that the worm compensates its simple anatomy with secretion of a novel toxin, or mixture of toxins, referred to by us as “phyllotoxins”. These are carried by mucus and delivered via repeated contact with the tip of the proboscis until the prey is relaxed or immobilised (reversibly). Proteolytic action permeabilises material to toxins and softens tissue to enable extraction by suction. The findings show that toxins are a major ecological trait and therefore play a key role in evolutionary success and diversification of Polychaeta, demonstrating also that understanding adaptative features may become the best showcase for novel animal toxins.

## Introduction

Chemical warfare is one of the most cost-effective strategies adopted by animals to defend against parasites, predators or to become predators themselves^1^. Biological toxins (biotoxins) can thus play an important ecological role and be regarded as adaptative features. Given the vastness of oceans and the ancient radiation of marine life, it is not surprising that the diversity of toxins may correlate with their immense biodiversity. Indeed, there has been a big effort to describe and catalogue novel toxins from marine eumetazoans. This enterprise is mostly motivated by biotechnological implications, which usually implies the very challenging endeavour to characterise the complex mixtures of proteins, small peptides and salts that comprise poisons and venoms^1^. Indeed, and despite much early promise, biotechnological applications of marine toxins seem disappointing^2,3^. At least in part, this issue results from the failure to understand the combined effect of the various elements in these mixtures. To this are added the difficulties in characterising toxins at the molecular level that derive from poor genomic annotation, a problem that hinders marine animal research in general. The present work is thus set upon the hypothesis that understanding the ecological role of biotoxins is the first step to understand the function and evolution of marine animal chemical weaponry.

Recent descriptions of novel toxins from Polychaeta and even the very first crustacean venom indicate that we may be merely facing a small part of the vast diversity of poisonous marine invertebrates^4,5^. In line with the trend to find novel biotoxins fortuitously, we recently came across a novel unknown proteinaceous toxin (≈40 kDa) secreted by a hitherto inconspicuous annelid, *Eulalia viridis* (Phyllodocidae), whose mucous secretions had a strong inhibitory reaction against the marine bacterium *Vibrio fischeri*^6^. The species inhabits rocky intertidal shores and has a clear preference for mussel beds^7^. One of the most interesting aspects of the animal’s ecology is that it is an active predator of much larger prey, particularly live mussels, barnacles and even Polychaeta (including other *E*. *viridis*). However, the species, as other members of the order, is devoid of jaws, relying solely on its powerful muscular proboscis for feeding^8^. We conjectured, then, that the worm uses toxins, referred to by us as phyllotoxins, as part of its preying strategy, enabling it to extract a portion of the prey’s soft body via suction.

The rapidly-expanding literature on marine animal toxins is making use of high-content screening molecular approaches to ascertain the nature of the proteinaceous materials in toxin mixtures^9^. Still, only in a few instances homology-based analyses have been able to produce convincing clues on the role of these secretions in interspecific interactions. Among these, Whitelaw *et al*.^10^ related the presence of chitinases in the scarcely known toxin mixtures from some cephalopods that predate on crustaceans (“cephalotoxins”). The authors argued that, considering the elevated contents of chitin in arthropod tissue (a glucose-derived polymer with analogous function to vertebrate collagen), this enzyme increases permeability to facilitate infiltration of neurotoxins. Understanding the composition of toxin mixtures is a challenge beyond the problem of genomic annotation, as it may be a function of environmental parameters such as diet^1,11,12^. It has been discovered that some animals can even produce different venoms for predation and defence, such as some cone snails and a few arthropods, like scorpions, for instance^1,13,14^. In any case, adaptative traits offer a solid ground to steer research and such is the motto of the present work. By combining ecological and toxicological endpoints, we aim at understanding the ecological role of toxins in the very particular behaviour of *E*. *viridis*, a discreet but resourceful organism that revealed itself to be a fierce predator.

## Results

### *E*. *viridis* feeding behaviour

**Figure 1:**
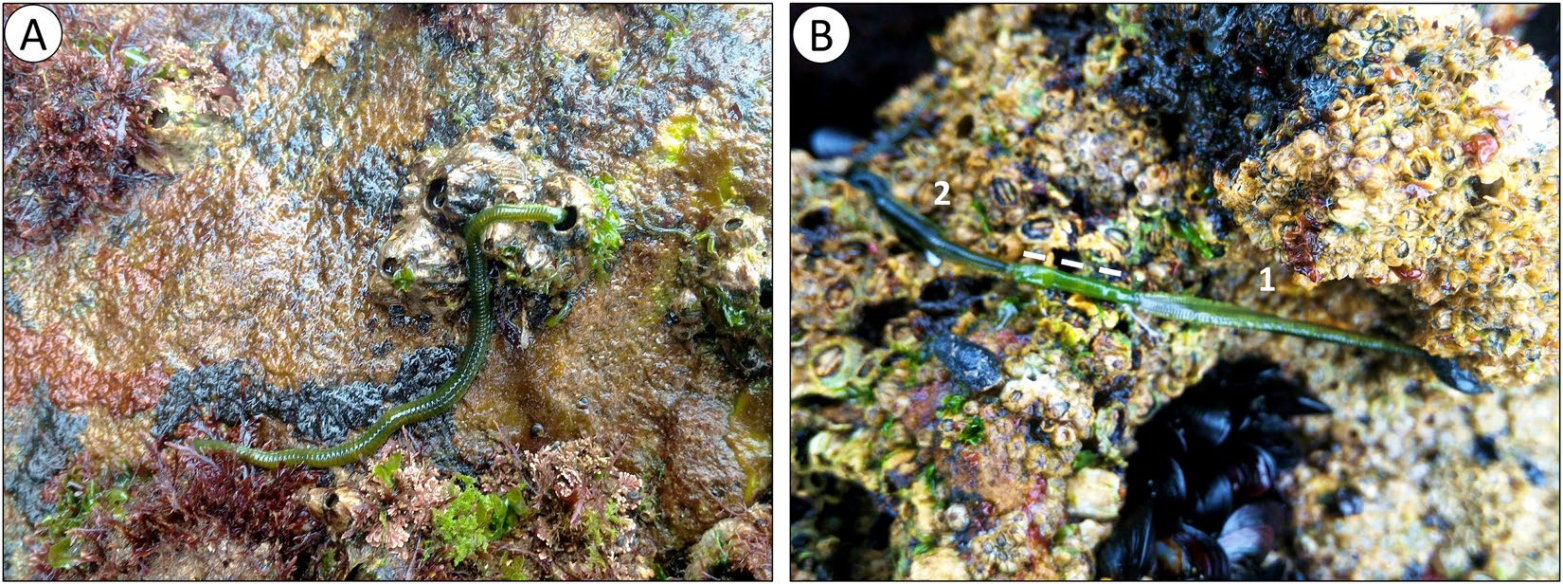
Feeding behaviour of *E*. *viridis* in its natural environment. (**A**) A worm inserting the proboscis between the plates of a barnacle. (**B**) A worm^1^ preying on another individual from the same species^2^ using its proboscis (dashed line).

In its preferential habitat, *i*.*e*. rocky intertidal mussel beds, in western Portugal, *E*. *viridis* was observed to be an opportunistic but active predator of a wide range of other invertebrates, with a preference for live mussels, barnacles and even other Polychaeta. The worm used the proboscis for sensing the environment during foraging and inserted it between the valves of mussels and plates of barnacles (Fig. 1). A clearer picture of the use of this organ was seen when attacking other annelids, as shown in the video and respective caption in Supplementary Information (SI). In the video it can be noted that contact with the target organism is localized and repeated via extension of the proboscis, accompanied by copious secretion of mucus. The prey tries to escape while becoming immobilised until shrivelling. At this point, *E*. *viridis* attempts to extract a piece of its flesh through the wound created at the contact area through suction movements with its proboscis.

### Toxin reactivity

**Figure 2:**
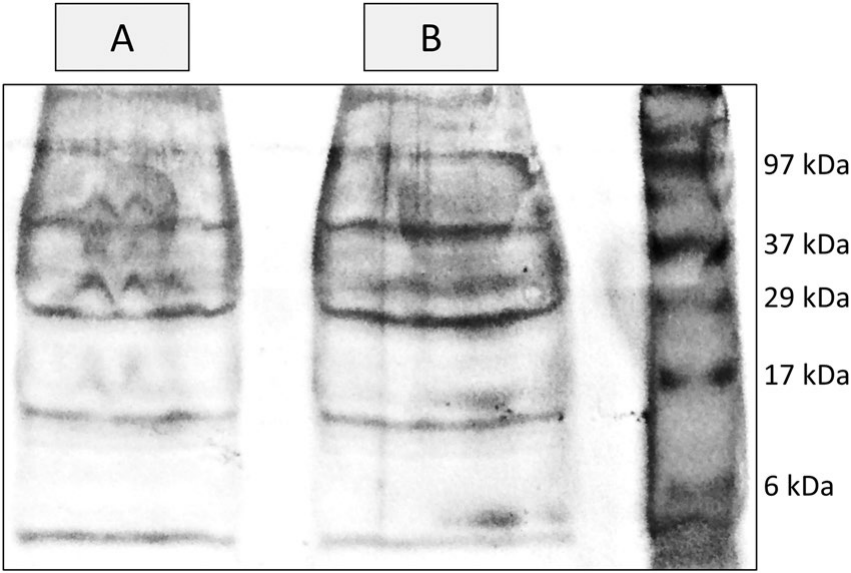
Protein signature of crude (**A**) and purified (**B**) extracts from mucosecretions, diluted to the same amount of total protein (1 mg/mL) in PBS and sterilised seawater, respectively, as visualised through SDS-PAGE (silver staining). Purification was done by ultrafiltration using a 3 kDa membrane, therefore removing salts and other small molecules. See Supplementary Fig. S1 for the original gel photograph.

The mucus was found to be moderately viscous, little adhesive and rapidly dissolved in natural seawater and other aqueous media. Purification by ultrafiltration isolated molecules larger than 3 kDa from secretions, yielding a multi-protein/peptide signature similar to that of crude mucus after removal of salts and other small constituents from secretions, with major bands between c.a. 6 and 40 kDa, which is compatible with toxins from marine invertebrates, such as conotoxins (Fig. 2). The bioreactivity of the crude secretions and purified peptides was then asserted using the standardised Microtox test, which determines toxicity from the inhibition of the luminescence of the marine bacterium *Vibrio fischeri*. The effect to bacteria was dose-dependent and the EC_50_ threshold at 5 min (the half-maximal reduction in bioluminescence) to the bacteria was 98 µg total protein per ml (95% confidence interval: 74–129) in seawater. The IC_50_ for purified peptides in PBS, at 5 min also, was higher (due to re-concentration of toxins as smaller compounds were removed) but within the same magnitude: 47 µg total protein per ml (38–56). The results confirm that the toxicity is chiefly conferred by the proteinaceous component of the mixture.

### Toxin mode-of-action *in vivo*

**Figure 3:**
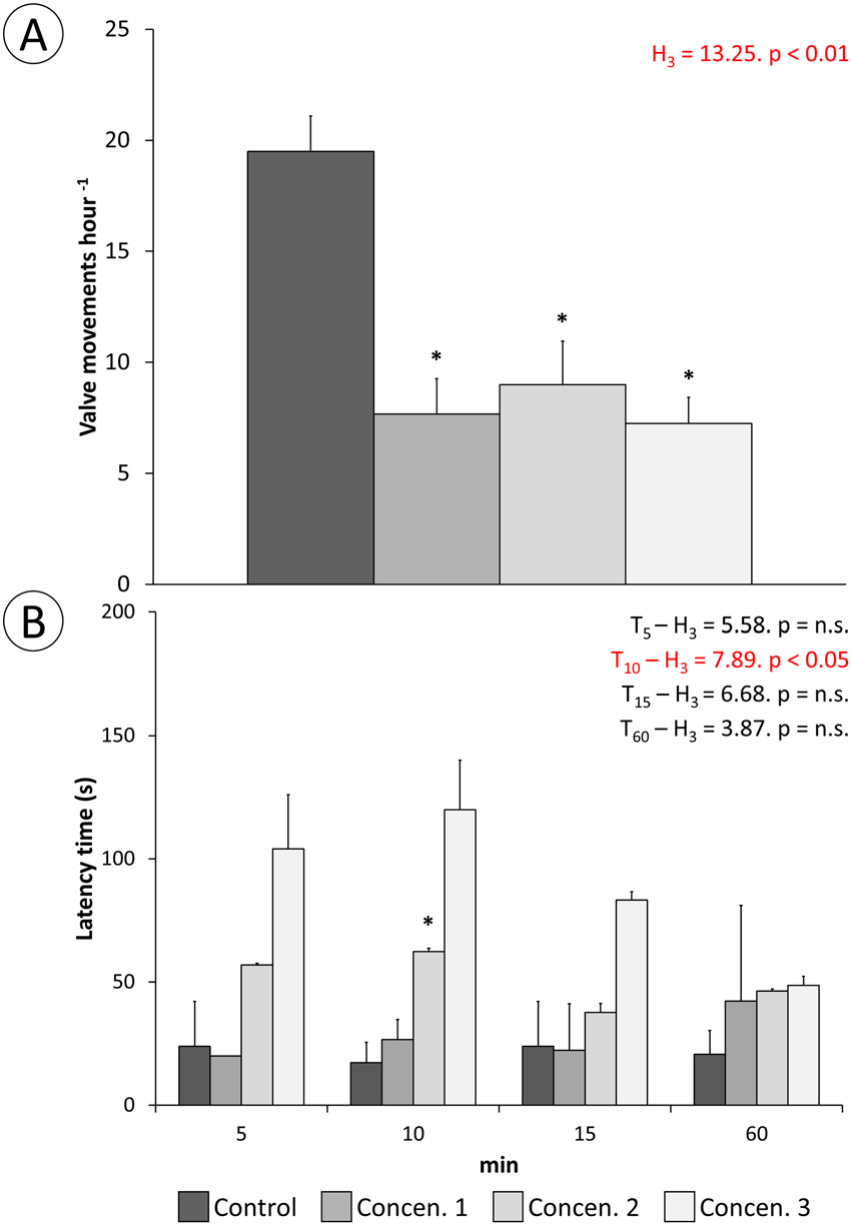
Behavioural responses in mussels exposed to toxic secretions (three concentrations) via intravalvar injection. (**A**) Valvar movement frequency. (**B**) Latency time to response (valvar re-opening) following stimulus (touch) in tested mussels treated with increasing concentrations of toxic secretions. The results are provided as means ± SEM. Statistical analyses were obtained with the Kruskal-Wallis ANOVA by ranks *H* for multiple comparisons. *Indicates significant differences to respective controls (Mann-Whitney *U*-test, *p* < 0.05).

**Figure 4:**
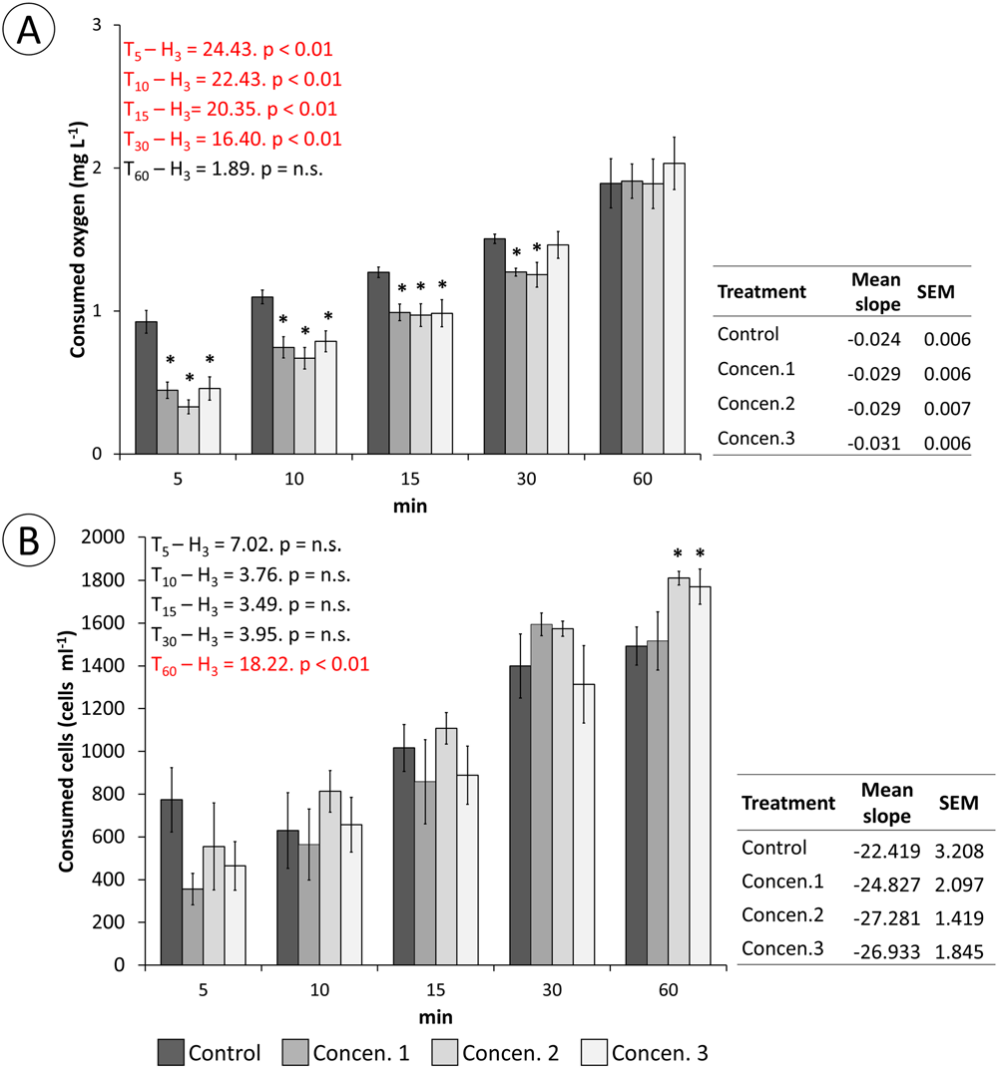
Physiological parameters in mussels exposed to increasing concentrations (Concen. 1 to Concen. 3) of toxic secretion. (**A**) Consumed oxygen. (**B**) Microalgae removal from water. Side panels show rates of removal (oxygen and alga cells) from water, per experimental treatment. The results are provided as means ± SEM. Statistical analyses were obtained with the Kruskal-Wallis ANOVA by ranks *H* for multiple comparisons. *Indicates significant differences to respective controls (Mann-Whitney *U*-test, *p* < 0.05).

**Figure 5:**
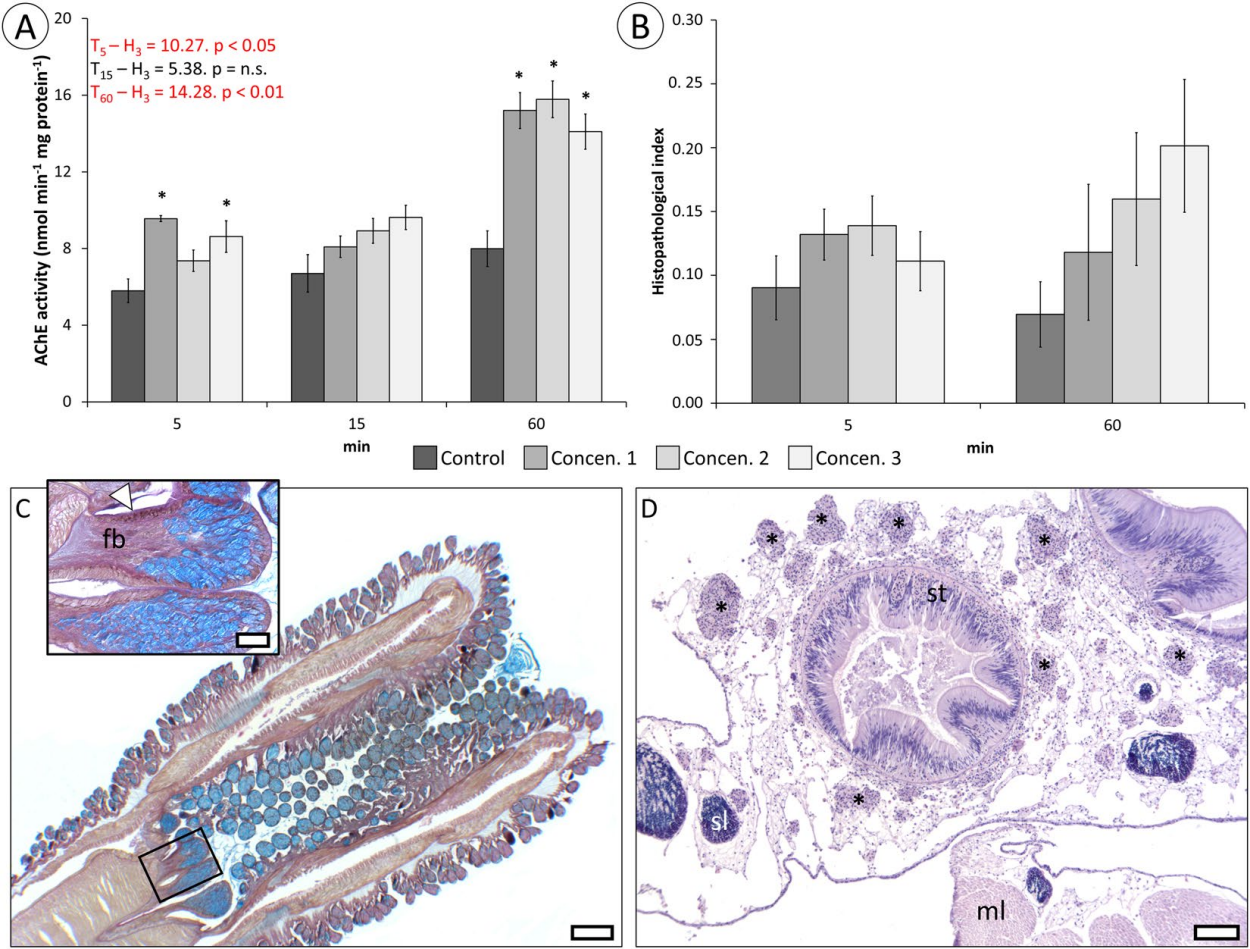
Neurochemical and toxicopathological effects in mussels exposed to increasing concentrations of toxin secretions (Concen. 1 to Concen. 3) at different timepoints. (**A**) Acetylcholine esterase (AChE) activity in adductor muscle. (**B**) Integrated (multi-organ) histopathological condition index. The results are provided as means ± SEM. Statistics were obtained with the Kruskal-Wallis ANOVA by ranks *H* for multiple comparisons. *Indicates significant differences to respective controls (Mann-Whitney *U*-test, *p* < 0.05). (**C**) Longitudinal section of the proboscis of *E*. *viridis* (not fully everted) highlighting toxin-delivery tentacles (box). Blueish cells are mucocytes, revealed by Alcian Blue dye in the tetrachrome stain. Inset: high-power magnification of toxin-delivery tentacles, highlighting serous cells were toxins and enzymes are produced (arrowhead) and bundles of connective and nervous fibres (fb). (**D**) Histological section across the visceral mass of mussels one hour elapsed after injection of the highest concentration of toxin (Concen. 3). Several foci of defence cells agglomerates (*) indicate mild inflammation, close to stomach (st) (H&E stain). sl: seminiferous lobe; ml: muscle. Scale bars: (**C**) 200 µm, inset 50 µm; (**D**) 200 µm.

Using live mussels as convenient model, the toxin’s mode-of-action was inferred through a series of bioassays conducted with crude mucous secretions diluted in sterilised seawater (whose reactivity was checked through the Microtox assay, as stated above) and applied via intravalvar injection to mimic administration of the toxin-containing secretions as the proboscis of the worm inserts itself in the mantle cavity. The frequency of valvar movements (opening or closure), determined during one hour after injection, was significantly reduced to about half in mussels exposed to the crude secretions, albeit without any evident dose-response (Fig. 3A). Also as a behavioural biomarker, increased latency time (elapsed time between touch and valve re-opening) was evident in treated animals, relatively to controls, particularly after 10 min, then showing recovery (Fig. 3B). These data agree with the reduction, followed by recovery, of two physiological biomarkers in exposed mussels, namely filtration rate (Fig. 4A), determined by microalga removal from water, and oxygen consumption (Fig. 4B). An interesting effect was noted regarding the previous, though, as the mussels clearly over-responded one hour elapsed after injection with the most concentrated form of the crude toxin, suggesting hormesis. The recovery is reflected by the steeper rates (slopes) of the two parameters in exposed animals resulting from augmented removal of algae and oxygen from water 60 min after injection. Similarly, no significant reduction in acetylcholinesterase (AChE) activity was observed in the adductor muscle of exposed mussels, but rather an increase, more obvious one hour after administration of crude secretion, once more without an evident dose-response (Fig. 5A).

Histopathological alterations in exposed animals, translated into a condition index, were scarce and chiefly related to increased inflammatory response in exposed animals. These alterations refer to focal infiltration of haemocytes in several tissues, with emphasis on the external portion of the digestion gland and adjacent mantle (*i*.*e*., the more exposed areas). Despite the trend to increase 1 h after exposure, the dissemination of alterations was variable, which made determining statistical significance difficult (Fig. 5B). Nonetheless, significant differences after 1 h of injection between the condition index of animals exposed to the highest dose (Concen. 3) and controls were dismissed with *p* = 0.06 (Mann-Whitney *U*-test). For guidance, in Fig. 5C is shown the location of the toxin delivery tentacles at the tip of the proboscis, which, in this case, is partially inverted. The dense agglomerates of mucous (blue) and serous (toxin-secreting) cells are evident, the latter of which line the entire base of tentacles. In Fig. 5D is exemplified the formation of focal agglomerates of defence cells (haemocytes) in an area of the digestive gland near the mantle of an exposed mussel (highest concentration of the toxin secretions).

**Figure 6:**
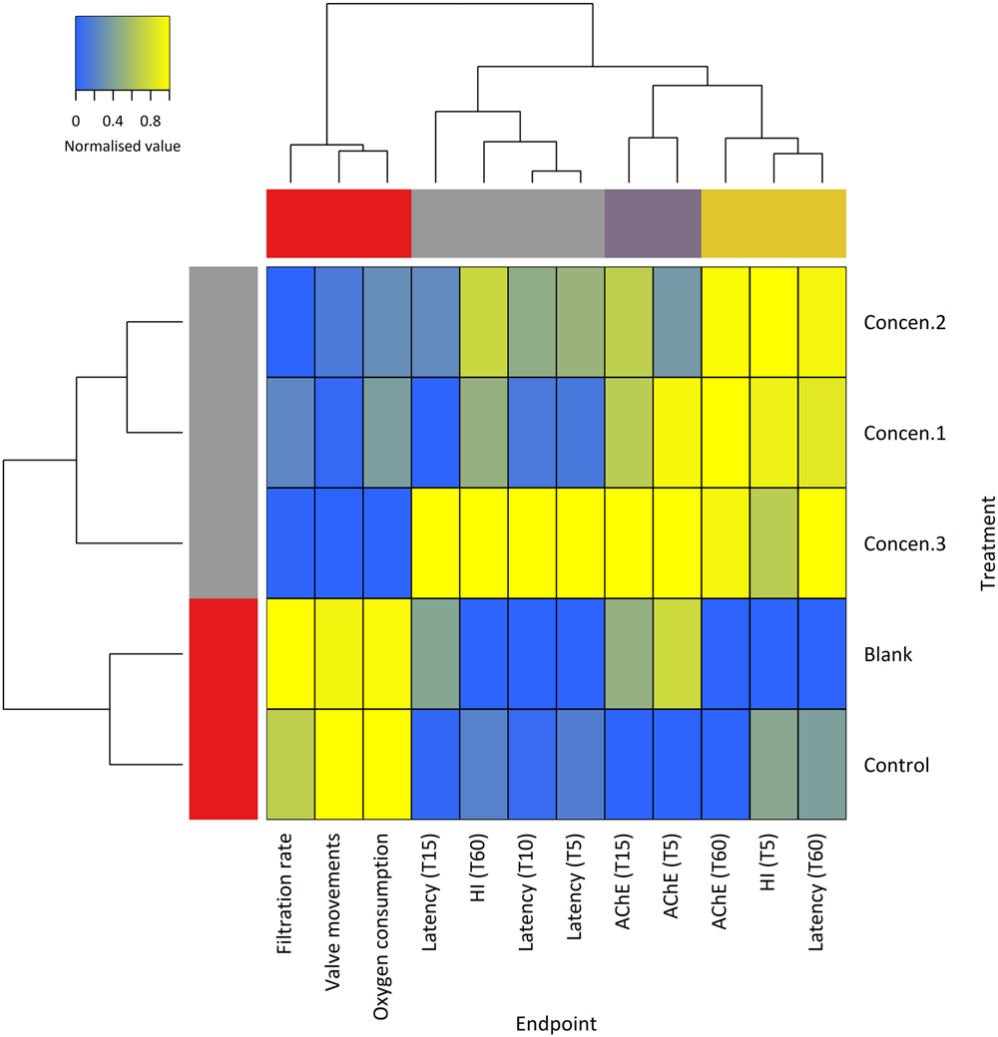
Heatmap and hierarchical clustering of normalised results obtained with complete linkage and Euclidean distances, aggregating all measured responses from mussels treated with toxins (separated by time after exposure). Hierarchical trees and colour bars indicate association between variables (up) and experimental conditions (side). Note the clustering between control and blanks, clearly separated from the treatments with toxins. The association between filtration, oxygen consumption and valve movements is also clear, which makes physiological sense, all of which being negatively affected by the toxin.

Hierarchical clustering of measured responses and effects during these bioassays (Fig. 6) allowed segregating experimental treatments in two major clusters. The fist comprised controls and blanks (*i*.*e*. animals injected with seawater only), the second all exposure treatments. Among the latter, exposure to the highest concentration (*i*.*e*. lowest dilution) stand out from the two preceding, with particular respect to delayed responsive behaviour (latency time) and histopathological alterations, especially after one hour of injection. In their turn, variables are segregated in two major clusters (upmost hierarchical tree), the first of which including physiologically-related variables, namely algae filtration, O_2_ consumption and valvar movement. These parameters were rapidly reduced by exposure to toxin secretions. The remaining responses correspond to effects that were enhanced by exposure, from AChE activity to histopathology and latency time, which thus showed an inverse pattern to that of the preceding cluster. The positioning of AChE parted from valve movements and related responses indicates that phyllotoxins are unlikely to have an inhibitory effect on this post-synaptic enzyme.

### Pathological aspects of direct contact with mucous secretions

**Figure 7:**
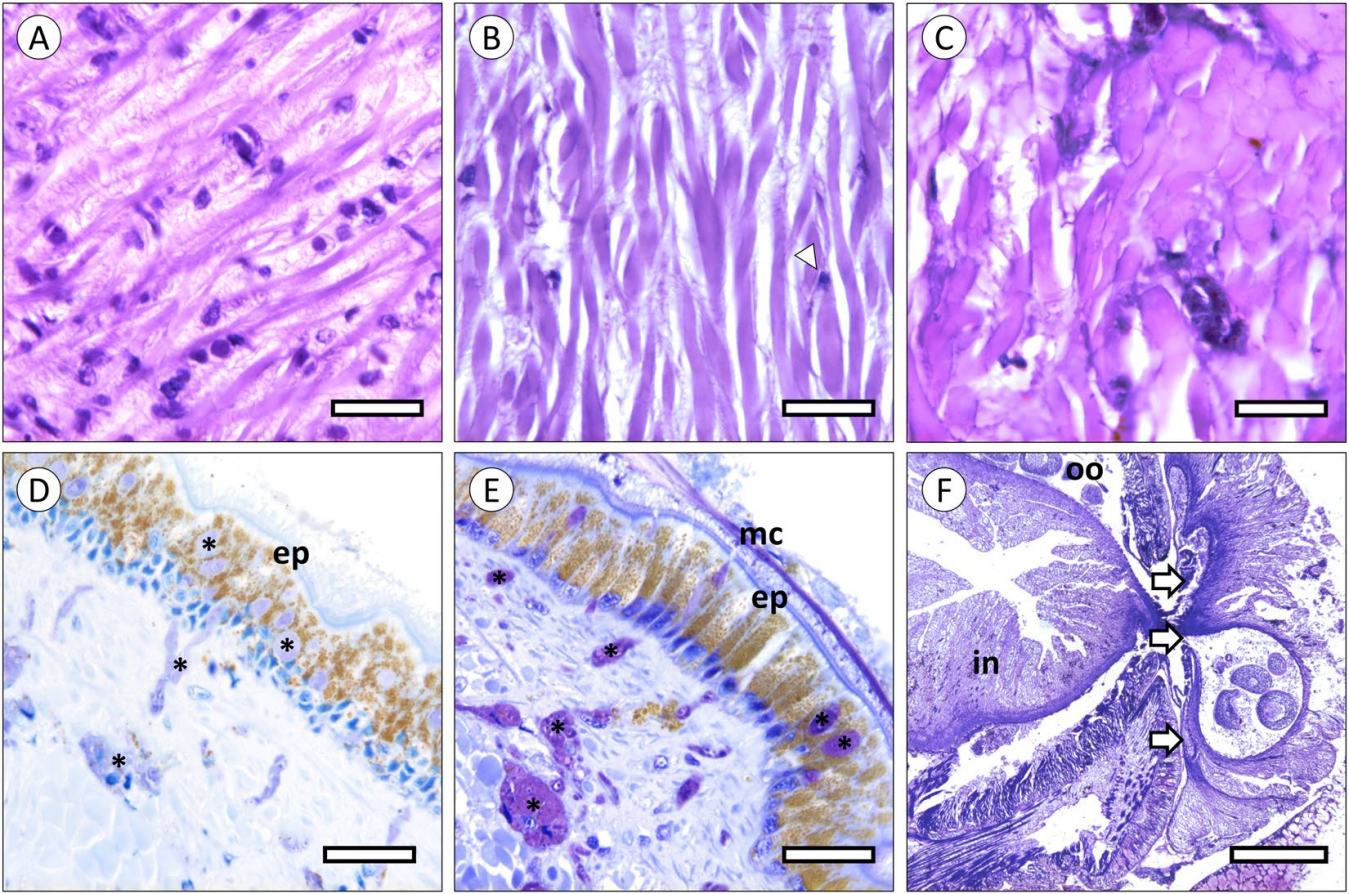
Histopathological evaluation of the effects of toxin in *E*. *viridis* mucus at the contact area with the crude secretions, in natural prey. (**A**–**C**) Muscle tissue from the foot of mussels (H&E). (**A**) Normal aspects of tissue. (**B**) Moderately affected muscle in a foot exposed *ex vivo* to the mucous secretions, showing disorganization of connective fibres and hyalinisation (digestion) of muscle fibres, which become more eosinophilic (pink). Note the abnormal aspect of nuclei (arrowhead), indicating early cell death. (**C**) Foot muscle of a live mussel exposed to crude mucus, revealing severe hyalinisation of muscle fibres. (**D**,**E**) Resin (semi-thin) sections of the edge of the foot (PAS-Toluidine). (**D**) Normal secretory epithelia and underlying connective tissue (unexposed animal). (**E**) Proliferation of secretory cells (*) and increased secretion. Note the layer of mucus (mc) atop the ciliated epithelium of the mussel (ep). Resin section (semi-thin) of the wound caused by an *E*. *viridis* onto a conspecific, in its habitat. (**F**) The toxic secretion partially digested the body wall of the prey, enabling suction of contents (arrows), up to the point where oocytes (oo), which mature in the coelom, can be observed being pulled out, together with a portion of the intestine (in). Scale bars: (**A**–**E**) 20 µm; (**F**) 400 µm.

As intravalvar injections likely dispersed of mucus and toxins, there was the need to ascertain the realistic effects resulting from the direct contact with mucous secretions. In order to mimic direct contact, we applied freshly collected mucus (from the proboscis) onto the soft tissue of whole live mussels or through *ex vivo* contact with freshly excised organs. The results showed more prominent histopathological alterations, comparatively to the previous assays. In Fig. 7A–C are shown sections from foot muscle, showing digestion of muscle fibres evidenced by myocyte hyalinisation, accompanied of disorganisation of connective fibres and apoptotic cells. The effects were stronger in whole-mussels than in *ex vivo* assays. Alterations in the glandular epithelium lining the mantle were also evident in the contact area (Fig. 7D,E). These alterations are chiefly related to increased secretory activity, as seen from the proliferation of granular cells suggests detoxification and increased production of protective glycoproteins, mucins included.

## Discussion

As *E*. *viridis* joins the ranks of toxin-secreting Polychaeta, it is shown that small body size and low level of organ differentiation, features shared by most extant protostomes, can be circumvented by chemical warfare as strategies for predation and defence. The aggregate effect of the cocktail of proteinaceous toxins, here referred as “phyllotoxins”, present in the mucous secretions of the worm are applied to the prey by repeated contact with the tip of the proboscis, where specialised tentacles are located, eventually causing (reversible) immobilisation while partially digesting the soft tissue. The absence of massive tissue digestion (recall Figs 5 and 7), indicates that the main function of enzymes in mucosecretions is not extracorporal digestion but a combination between permeabilization to toxins and tissue softening to facilitate extraction by suction, as shown in Fig. 7F. It must be noticed that *E*. *viridis* is not only able to extract and ingest large portions of soft tissue but also entire prey, including conspecifics, as shown in our previous work on the species’ digestive function^15^. This ability reiterates the convenience of secreting immobilising or relaxing toxins. It must be noted, however, that the complexity of secretions containing toxins, plus the impossibility of neutralising noxious substances only, hinders establishing direct cause-effect relationships. Despite the aggregate evidence for *Eulalia* toxins, as for other Phyllodocida, which is based on multiple endpoints, there are recent indications that bivalves can respond to undisclosed waterborne chemical clues from potential predators. Sensing these clues can lead to various defensive changes, from the reduction of metabolic and filtration rates to the thickening of shells in a predator-laden habitat^16–18^. It is thus possible that these chemical clues may have contributed to the lowering of physiological and related behavioural parameters such as gas exchange, filtration and valvar movements, even though they cannot explain *per se* the observed toxicopathological effects and how *Eulalia* can penetrate through the valves. In any case, the complexity of toxin-bearing animal secretions mandates some caution when inferring specific effects.

Despite the little work done, so far, on Polychaeta, some authors have already reported toxins from these animals. The toxicology and the ecological role of these substances remains, nonetheless, little understood. Among these, the best-known cases are nereistoxin, arenicin and glycerotoxin^19–21^. Nereistoxin, in particular, is a non-proteinaceous neurotoxic alkaloid that originates from the salivary gland of *Lumbrinereis heteropoda*. The role of this toxin remains elusive, as it is known by its insecticide properties with neuromuscular effects that have been associated to its metabolites and not to the parental compound or compounds^22,23^. Nereistoxin is nowadays considered one of the most promising basis to develop “ecologically-sustainable” insecticides. Glycerotoxin, in its turn, refers to a well-known high molecular weight (c.a. 320 kDa) protein from the venom glands of bloodworms (*Glycera tridactyla*), which belong to Phyllodocida just as *Eulalia*. Even though recent works suggest the neurotoxicity of glycerotoxin based on molecular data^21^, the first descriptions of *Glycera* venom glands plus the successful isolation and testing of glycerotoxin isoforms as neurotoxins already date from the 1960s to the 1980s (see Schenning *et al*.^24^, plus Bon *et al*.^25^, and references therein). Another example, Arenicin, however, pertains to antimicrobial peptides produced in the coelomocytes of the marine Polychaeta *Arenicola marina* (lugworm), a burrower worm that, unlike the former, is not a predator^19^.

Being able to immobilise prey, even if partially or temporarily, is always an advantage for a predator. However, it is of particular importance for those that cannot physically out-compete their targets, such as *E*. *viridis*. There are several uncanny adaptations for the purpose. It is the case, for instance, of the modification of one of the claws of the snapping shrimp *Alpheus heterochaelis*, whose clacking creates an air bubble that pops violently, producing a strong sound that stuns prey (or keeps attackers and competitors at bay), including much larger fish^26^. Chemical stunning seems to be more common, though, and likely more cost-effective. Existing data on marine invertebrate neurotoxins, particularly from *Conus*, support this premise but attempts to validate these occurrences as adaptative features, which involves understanding the mode-of-action of whole venoms onto ecologically-relevant targets, are scarce. In *E*. *viridis*, the immobilising effect of the toxin is reversible and not immediate. In fact, *in situ* observations and the endpoints illustrated in Figs 3, 4 and 5 suggest a peak of effects between 5 and 10 min, followed not only by recovery and even over-response, most likely a hormetic effect to compensate non-lethal challenge, *i*.*e*. a beneficial over-response to reduced toxicological stress (see Calabrese *et al*.^27^). It must be noticed that the mussel bioassays may underestimate the full potency of phyllotoxins, as they do not mimic the repeated, direct contact, promoted by the worm *in situ*. This explains lower histopathological effects in tested mussels, comparatively to the *ex vivo* assessment (Fig. 7A–E), which was done by direct contact of mucus onto tissue rather than intravalvar diffusion, and the consequences to the captured annelid shown at Fig. 7F. However, in either case, histological alterations pertain mostly to fibrous tissue, which indicates the need to soften and permeabilise the material prior to extraction of large pieces and not to uphold extracorporal digestion, as in maceration.

Comparatively, conotoxins are fast-acting and many are lethal. Among well-known lethal toxins, tetrodotoxin (TTX), which has been detected in several marine organisms such as blue-ringed octopus and pufferfish, is one of the most powerful neurotoxins^28^. Additionally, potent and fast-acting toxins are usually injected, which allocates mixtures of conopeptides and other neurotoxins into the category of “venoms”^29^. However, there is a huge variety of conotoxins, by far the best-studied natural marine toxic compounds, the vast majority of which is studied from recombinant forms and not in its native forms, which also means that the effects on prey are not understood (see Akondi *et al*.^30^, for a review). Altogether, our data suggest that phyllotoxins have a mode-of-action *in vivo* distinct of most fast-acting neurotoxic conopeptides and similar, many of which target voltage-gated ion channels directly. In addition, the failure to correlate “behavioural” parameters such as valve movements and latency time with AChE suggests that the toxin does not interfere with this serine hydrolase directly and that its increase is indeed the consequence of the over-response mentioned earlier. The absence of any form of complex gland of venom-injecting apparatus in *E*. *viridis* is in accordance with the animal’s behaviour and toxin administration via mucous secretion from the tip of the proboscis, where specialised tentacles are located. Thus, as the toxins are not delivered through a wound, they fall within the proposed category of “toxungen”^29^. This form of delivery is coupled with repeated contact with indiscriminate soft-bodied prey, which may range from mussels to other Polychaeta. Still, while contact with annelids and even with some gastropods (like *Patella* spp.) can be relatively conspicuous and appears to be a relatively simple process, the way how the worm penetrates valves of mussels and plates of barnacles is more difficult to record and to explain. Nonetheless, the existence of the toxin *per se* sheds light on the process. Rovero *et al*.^31^ suggested that dogwhelks (*Nucella* spp.) can penetrate barnacles and even between the valves of bivalves using its proboscis as an alternative behaviour to the more common process of drilling through the shells of mussels, using its radula, by secreting an unknown immobiliser (“relaxant”) to facilitate insertion. Given the form and function of the proboscis, it seems evident that *E*. *viridis* operates in a similar way, especially considering that the animal is not equipped with structures able to force against the powerful adductor muscles of bivalves^32^ or drill through shells and plates, for instance.

As in the case of cephalotoxins, it has been hypothesised that the presence of specific enzymes in toxin mixtures may facilitate the infiltration of neurotoxins^10^. In *E*. *viridis*, these enzymes seem to have the function of partially digesting tissue to allow extraction. The effects of the secreted substances appear to be the digestion of muscle fibres and connective tissue. Although some evidence for cell death (likely apoptosis due to condensation and blebbing of nuclei) having been registered (Fig. 7B,C), it is not possible to ascertain whether this is a direct effect of some specific pro-apoptotic compound or due to action of proteinases, many of which are known to favour, if not trigger, programmed cell death, such as cysteine and serine peptidases^33^. Still, this issue is not yet well understood in invertebrates. In any case, the effect is sufficiently potent to perforate the body wall of other annelids and pull, through suction, the contents from the coelomic cavity, as well as the remnants from the partially digested musculature, as clearly illustrated in Fig. 7F. The combination between powerful suction, immobilising toxins and proteolytic activity thus maximises the predatorial abilities of the worm, in spite of the reduced complexity and differentiation so common in protostomes. These features make it a highly efficient predator in its environment, not just against immobile prey like barnacles and bivalves, but also against its immediate rivals, such as other predatorial Polychaeta, including its conspecifics.

## Methods

### Mucotoxin harvesting and characterisation

Adult worms (*c*.*a*. 5–10 cm length) were sampled by hand (*n* ≈ 300) during low tide at rocky beach in W Portugal (38°41′42″N; 09°21′36″W) and maintained in the laboratory in a microcosm environment. Crude mucous secretions were harvested by gentle mechanical stimulation using blunt-tipped plastic tweezers. The mucus samples were pooled and centrifuged to remove solid deposits (5000 × *g*, 4 °C, 5 min) and stored at −80 °C until further analyses. To verify the proteinaceous nature of toxins, an aliquot of the mucus was subjected to ultrafiltration with using 3 kDa Amicon spin column filters (Merck Millipore) after preliminary filtration through cellulose acetate filter (0.22 µm). Dulbecco’s phosphate-buffered saline (PBS), pH 7.4 was employed vehicle to assess reactivity in physiologically-compatible media. The proteinaceous nature and toxicity of purified and crude secretions was assessed by sodium dodecyl sulphate polyacrylamide gel electrophoresis (SDS-PAGE) and the standardised Microtox test, respectively^6^. Total protein, determined with a NanoDrop 2000 apparatus (Thermo Fisher), was used to indicate concentration of toxins.

### Experimental assessment of toxin mode-of-action *in vivo*

Mussels, *Mytilus* sp. (4.5–5.5 cm shell length), were hand-collected between February and April 2017 from a clean rocky intertidal area in W Portugal as well. Crude secretions for testing were diluted in filtered and autoclaved seawater. The doses (concentrations) are designated as Concen. 3 (highest −1 mg/mL total protein, determined as above), Concen. 2 (0.5 mg/mL) and Concen. 1 (lowest −0.25 mg/mL). The animals were subjected to intravalvar injection once with each dose (0.6 mL). Controls (seawater only) were included, as well as blanks (no injection). Several independent assays were conducted in order to address multiple endpoints. The animals were collected at several time-points between 5 and 60 min after injection, depending on endpoint. The frequency of valve movements (opening or closure) was quantified from video analysis. Six biological replicates were analysed for behavioural endpoints (n = 6). Latency time was determined from stimulus (tapping) to valve re-opening. Alterations to physiology (n = 3) were determined from oxygen consumption using a Multiline 340i/SET electrode (WTW, Germany), and microalga cell (specially cultured *Tetraselmis suecica*) removal using a Multisizer 3 Counter (Beckman Coulter). Acetylcholine esterase (AChE) activity was determined in the adductor muscle of mussels according to the method developed by Ellman *et al*.^34^, modified for microplates. These results are provided as nmol hydrolysed substrate min^−1^ mg protein^−1^. Histopathological alterations were determined in whole soft-body of mussels (n = 6), fixated in Davidson’s solution and embedded in Paraplast. Sections (5 µm) were stained with haematoxylin and eosin (H&E) and the tetrachrome procedure described by Costa and Costa^35^. Semi-quantitative histopathological condition indexes were obtained according to the method described by Costa *et al*.^32^, adapted to mussels by Cuevas *et al*.^36^. Briefly, the procedure is based on the product between dissemination (from 0 – absent to 6 – diffuse) and biological significance (1 – lowest severity to 3 – highest). The histopathological alterations selected for the estimation of indexes (following preliminary observations) were lipofuscin aggregates, haemocytic infiltration and diffusion of brown cells, all of which have a biological significance of 1^32,36^. Accuracy was checked by blind reviews. The methodology produces an integrated histopathological condition index that ranges between 1 (maximum predicted histopathological condition) and 0. Indexes were obtained per individual and integrated measurements from visceral mass, gills, nephridium and gonad.

### Toxicopathological effects under natural conditions

In order to simulate direct contact with the toxic secretions, freshly-collected whole mucus was applied directly onto soft tissues live mussels or freshly excised organs (*ex vivo* assessment) (n = 2). Tissue samples were then processed for histopathological analyses, to which was added analyses of prey collected from the natural habitat after being preyed by *E*. *viridis*.

### Statistical analyses

The normality and homoscedasticity of data were analysed through Kolmogorov–Smirnov’s and Levene’s tests, respectively. Considering the invalidation of at least one of the assumptions, non-parametric statistics were employed, namely the Kruskal-Wallis ANOVA-by-ranks *H* was applied for multiple comparisons (testing of effects) and the Mann-Whitney *U*-test for comparisons between experimental treatments and controls. Cluster analyses were also carried out for grouping tested variables. Statistics were performed with R 3.3x^37^ and the significance level was set at 0.05 for all analyses.

## Electronic supplementary material

Supplementary information

SI Video
