# Supplementary material for: Explorations on the ecological role of toxin secretion and delivery in jawless predatory Polychaeta
Source: Sci Rep. 2018 May 16;8:7635. doi: 10.1038/s41598-018-26031-1 (PMC5955894; doi:10.1038/s41598-018-26031-1)
Supplement: Supplementary file 1 — Supplementary information [file 41598_2018_26031_MOESM1_ESM.pdf]

Explorations on the ecological role of toxin secretion and delivery in jawless predatory Polychaeta

Supplementary Information

Cuevas, N.[1], Martins, M.[2,3], Rodrigo, A.P.[1,2], Martins, C.[1,2], Costa, P.M.[1,*]

[1]UCIBIO – Research Unit on Applied Molecular Biosciences, Departamento de Ciências da Vida, Faculdade de Ciências e Tecnologia da Universidade Nova de Lisboa, 2829-516 Caparica, Portugal

[2]MARE – Marine and Environmental Sciences Centre. Departamento de Ciências e Engenharia do ambiente, Faculdade de Ciências e Tecnologia da Universidade Nova de Lisboa, 2829-516 Caparica, Portugal

[3]UCIBIO – Research Unit on Applied Molecular Biosciences, Departamento de Química Faculdade de Ciências e Tecnologia da Universidade Nova de Lisboa, 2829-516 Caparica, Portugal

Contents

Video caption .... P. 2

Figure S1. Original SDS-PAGE gel from mucosecretion extracts .... P. 3

[*] Corresponding author

Tel. +351 212 948 300 ext. 11103

pmcosta@fct.unl.pt

Video Caption

The video shows *Eulalia viridis* preying on another Polychaeta in its natural habitat. The first part shows how the worm uses the tip of its muscular proboscis (eversible pharynx) to deliver the toxin, which involves the copious secretion of mucus. The second part shows the already partially immobilised prey, highlighting the extension of the proboscis at close range. The minute toxin-delivery tentacles at the tip of the organ (refer to Fig. 5C) are clearly visible. The prey already exhibits a previous wound caused by its attacker. The video is shown in real time.

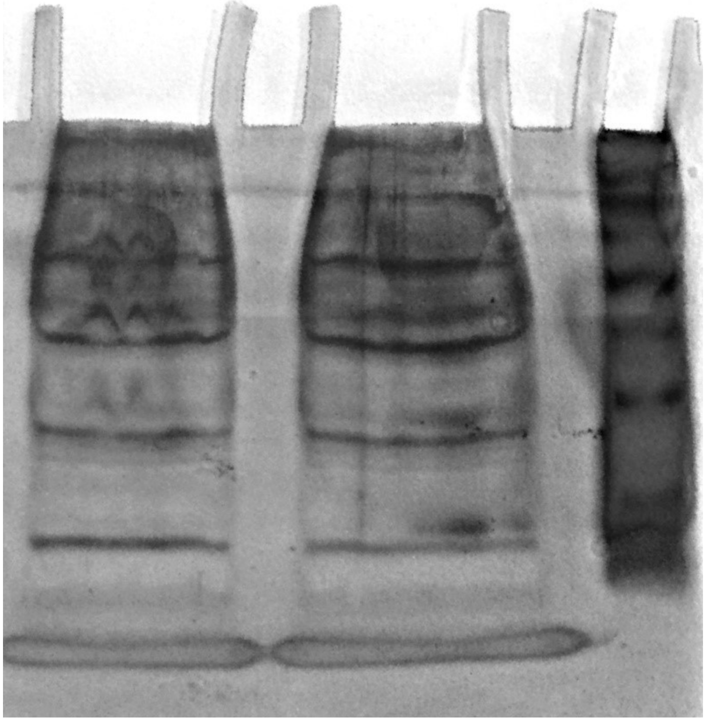

Figure S1. Original SDS-PAGE gel photograph of Fig. 2 (silver staining), showing the similar peptidic signature between crude (left) and purified (right) mucosecretions, diluted to the same amount of protein (1 mg/mL). The purified extract was prepared in sterilised PBS. Refer to Fig. 2 caption for further details.
